# Patient-Clustered Analysis of Recorded Insertion Torque in 101 MultiNeO Titanium Dental Implant Placements: An Exploratory Retrospective Cohort Study

**DOI:** 10.3390/ma19163560

**Published:** 2026-08-21

**Authors:** Katarzyna Wieczorek, Adrian Wasilewski, Bartłomiej Szwed, Weronika Nicpoń, Natalia Szymkowiak, Grzegorz Hajduk, Mansur Rahnama-Hezavah, Michał Łobacz

**Affiliations:** Chair and Department of Oral Surgery, Medical University of Lublin, ul. Chodźki 6, 20-093 Lublin, Poland; adrian.wasilewski14@gmail.com (A.W.); szwedbart@gmail.com (B.S.); nicponweronika9@gmail.com (W.N.); nataliaszymkowiak4@gmail.com (N.S.); esso53@wp.pl (G.H.); michal.lobacz@umlub.edu.pl (M.Ł.)

**Keywords:** dental implants, insertion torque, primary stability, generalized estimating equations, MultiNeO, titanium alloy, anatomical site, retrospective cohort

## Abstract

Primary implant stability arises from mechanical engagement at placement, and insertion torque is not a direct measure of osseointegration. This retrospective single-centre study included 101 MultiNeO titanium implant placements in 55 adults treated between April 2023 and January 2026. Four experienced operators placed implants subcrestally using a W&H Implantmed SI-1023, a bone-resistance-adapted drilling protocol, and a programmed target of 35 N·cm. Associations with recorded torque were assessed using a patient-clustered Gaussian generalized estimating equation adjusted for sex, age, anatomical sector, implant length and diameter, hard-tissue augmentation, and placement timing. The mean torque was 31.63 ± 5.57 N·cm; the median was 35 N·cm (Q1–Q3, 30–35). Recorded torque was 3.25 N·cm higher for implants placed in women than for those placed in men (95% CI 1.20–5.31; *p* = 0.002). Relative to maxillary anterior/premolar sites, mandibular molar sites showed higher torque (β = 4.02; *p* = 0.001), whereas mandibular anterior/premolar sites showed lower torque (β = −5.41; *p* = 0.013). Implant length had a modest adjusted association (β = 0.93 N·cm/mm; *p* = 0.022); age, diameter, augmentation, and placement timing did not. The concentration of recorded values at 35 N·cm reflects a clinical target rather than the device limit. These exploratory associations do not establish an intrinsic sex effect or predict osseointegration, and residual confounding due to unmeasured bone characteristics remains possible.

## 1. Introduction

Osseointegration is a biological process that develops after placement, whereas primary stability denotes the mechanical fixation present at the end of surgery [1,2,3]. Insertion torque reflects rotational resistance during implant advancement and final seating. Although it is useful intraoperatively, it captures only one aspect of primary stability and correlates imperfectly with resonance-frequency measurements or later biological stability [3,4]. Consequently, insertion torque should not be used as a synonym for osseointegration or as a stand-alone predictor of long-term success.

The recorded torque is generated by an interaction among implant material, macrogeometry, the dimensions of the prepared osteotomy, cortical and trabecular architecture, and the insertion protocol. Taper, thread pitch and depth, coronal microthreads, cutting flutes, surface topography, implant diameter, and implant length can all change the mechanical response at the implant–bone interface [5,6,7,8,9,10,11,12]. Bone density and cortical thickness may be at least as important as implant dimensions, and the effect of one variable may become apparent only after adjustment for the others [7,8,9,10,11,12,13,14,15].

Whether patient sex is independently associated with primary implant stability remains uncertain. Sex-related differences in skeletal size, cortical and trabecular remodelling, hormonal status, and age distribution provide biologically plausible hypotheses, but published implant studies have produced inconsistent results [16,17,18,19,20]. Apparent differences may also arise from the distribution of implant sites, implant dimensions, treatment indications, or repeated observations from the same patient. An analysis that ignores patient clustering cannot separate these possibilities reliably.

Placement timing introduces another layer of heterogeneity. Immediate implants obtain fixation from the residual socket walls and from apical, palatal, or lingual bone beyond the socket, whereas delayed implants are placed in a healed ridge. Neither approach is intrinsically associated with higher insertion torque; the result depends on socket morphology, available native bone, osteotomy preparation, implant geometry, and case selection [21,22,23,24,25,26].

The scientific contribution of a retrospective cohort does not derive from the number of implants alone. In the present study, all placements involved one implant family with defined alloy, surface, connection configurations, and macrogeometry, and the analysis accounted for repeated implants within patients. This provides an exploratory estimate of the relative contribution of patient-, site-, procedure-, and implant-related variables under routine clinical conditions. The primary objective was to identify variables independently associated with recorded insertion torque. A secondary objective was to determine whether the unadjusted association with sex persisted after adjustment for anatomical sector, age, implant dimensions, augmentation, and placement timing.

## 2. Materials and Methods

### 2.1. Study Design, Setting, and Ethical Approval

This retrospective, single-centre cohort study used routinely recorded clinical data from the Chair and Department of Oral Surgery, Medical University of Lublin, Poland. The source dataset contained 101 implant placements performed between 4 April 2023 and 21 January 2026. All observations in the source dataset with complete values for the analytical variables were included; no a priori sample-size calculation was performed. Reporting was guided by the STROBE statement [27].

The study was conducted in accordance with the Declaration of Helsinki and was approved by the Institutional Ethics Committee of the Medical University of Lublin (protocol KE-0254/248/11/2023; 23 November 2023). The approval remained valid throughout the entire record-inclusion period, including records through 21 January 2026. Informed consent was waived because the study used anonymized routine clinical records and involved no additional intervention.

### 2.2. Participants, Data Linkage, and Eligibility

Records were linked at the patient level before de-identification, and each individual was assigned an anonymized study identifier. The final dataset comprised 55 unique adults and 101 implant-level observations. The same patient identifier was retained for all placements in that patient, allowing the dependence between repeated observations to be addressed statistically.

Eligibility required an age of at least 18 years and complete documentation of sex, date of birth, date of placement, implant site, implant length, implant diameter, placement timing, additional regenerative procedures, and insertion torque. The available file did not contain a screening log for records considered before construction of the analytical dataset; therefore, the number of earlier exclusions and the reasons for them could not be reconstructed. Postoperative outcome was not used as an exclusion criterion.

Smoking, diabetes, osteoporosis, antiresorptive treatment, systemic corticosteroid use, periodontal history, and other systemic conditions were not recorded in a sufficiently standardized case-level form and were therefore unavailable for analysis. These factors were treated as potential sources of residual confounding rather than as assumed exclusion criteria.

### 2.3. Implant System and Material Characteristics

All placements used Alpha-Bio Tec MultiNeO implants (Alpha-Bio Tec Ltd., Modi'in-Maccabim-Reut, Israel). According to the operative protocol, Conical Narrow Connection (CHC) implants were used for diameters of 3.2 and 3.5 mm, while Conical Standard Connection (CS) implants were used for diameters of 3.75 mm and larger. Before the final analysis, discrepant dimensional entries were checked against the source implant records and the manufacturer’s size matrix. The verified dataset contained implant lengths of 8, 10, 11.5, and 13 mm and diameters of 3.2, 3.5, 3.75, 4.2, and 5.0 mm. MultiNeO implants are manufactured from Ti-6Al-4V ELI and feature the NanoTec surface, produced by sandblasting and double thermal acid etching. Their macrogeometry includes a slightly tapered body and core, variable threads, coronal microthreads, cutting flutes, platform switching, and a narrow centring apex [28,29].

### 2.4. Osteotomy Preparation, Implant Insertion, and Torque Recording

Procedures were performed by four implant surgeons, each with experience exceeding 3000 implant placements. The implants were inserted subcrestally; the exact subcrestal depth was not stored as a quantitative variable. Osteotomies were prepared under irrigation using the manufacturer’s MultiNeO protocol, with the final drill selected according to implant diameter and tactile intraoperative assessment of bone resistance. Lower-density bone was underprepared, while dense cortical bone received the larger final or cortical drill recommended by the manufacturer [30].

For CHC implants, the manufacturer’s final preparation targets for diameters of 3.2 and 3.5 mm were, respectively, 2.0 and 2.4 mm in type IV bone, 2.8 and 3.0 mm in type II/III bone, and 3.0 and 3.2 mm in type I bone. For CS implants of 3.75, 4.2, and 5.0 mm, the corresponding final targets were 2.8, 3.2, and 3.65 mm in type IV bone; 3.2, 3.65, and 4.1 mm in type II/III bone; and cortical preparation to 3.65, 4.1, and 4.8 mm in type I bone. Sequential drilling began with a 2.0 mm pilot drill, and the final straight drill was kept 3 mm short of the implant length where specified in the manufacturer’s chart; the equivalent step-drill sequence could be used throughout the full implant length [30]. Bone quality guided the preparation but was not retained as a standardized D1–D4 variable, so it could not be included in the statistical model.

Implants were inserted mechanically using an Implantmed SI-1023 unit (W&H Dentalwerk Bürmoos GmbH, Bürmoos, Austria) with the EM-19 LC micromotor and WS-75 L 20:1 implantology contra-angle. The dedicated P4 implant-placement programme operates at 15 rpm in the forward direction and 30 rpm in reverse, and the system provides automatic torque control over a technical range of 5–80 N·cm [31]. The programmed clinical target during insertion was 35 N·cm. This value was selected as the unit’s routine target because the manufacturer’s educational guidance describes 35 N·cm as an ideal insertion torque and as the minimum indicated for immediate placement or immediate loading [32]. Within the clinical protocol, attainment of this target preserved the options of submerged healing, transgingival healing with a healing abutment, or—when anatomical, prosthetic, and occlusal criteria were also satisfied—immediate provisionalization with a temporary crown. Reaching 35 N·cm did not, by itself, mandate immediate loading. The study outcome was the insertion-torque value displayed during final machine seating and entered in the clinical record. Values were documented in 5-N·cm increments and ranged from 15 to 40 N·cm; the five records containing 40 N·cm were retained as documented. Thus, 35 N·cm was a protocol target rather than a hardware ceiling.

### 2.5. Variables and Anatomical Classification

Recorded insertion torque in N·cm was the dependent variable. Candidate predictors were sex as recorded in the clinical file, age at placement, anatomical site, implant length, implant diameter, hard-tissue augmentation, and immediate versus delayed placement. Immediate placement was defined as insertion at the extraction visit; all other placements were categorized as delayed. Hard-tissue augmentation included guided bone augmentation, autogenous grafting, and transcrestal or lateral sinus-floor elevation. One isolated connective-tissue graft was not classified as hard-tissue augmentation.

Detailed FDI regions were grouped as 11–15, 16–18, 21–25, 26–28, 31–35, 36–38, 41–45, and 46–48. To avoid an overparameterized adjusted model, these were aggregated before modelling into four sectors: maxillary anterior/premolar, maxillary molar, mandibular anterior/premolar, and mandibular molar. The eight-region analysis was retained for descriptive and unadjusted post hoc comparisons.

### 2.6. Statistical Analysis

Categorical variables are reported as counts and percentages. Numerical variables are summarized as mean ± standard deviation, median with the first and third quartiles (Q1–Q3), and range. Unadjusted two-group comparisons used the Mann–Whitney U test, and comparisons across more than two groups used the Kruskal–Wallis test. Continuous age, implant length, and implant diameter were examined using Spearman rank correlation. Pairwise comparisons after a significant Kruskal–Wallis test used Dunn’s procedure with Holm adjustment for multiplicity. All tests were two-sided, and *p* < 0.05 was considered statistically significant. No alternative global significance threshold was prespecified as part of a documented multiplicity strategy.

The primary adjusted analysis used a Gaussian generalized estimating equation with an exchangeable working correlation structure and robust standard errors, clustering observations by patient [33]. The model included sex, age per 10-year increment, anatomical sector, implant length, implant diameter, hard-tissue augmentation, and placement timing. Maxillary anterior/premolar sites served as the anatomical reference. No interaction terms were fitted because the sample contained only 55 patient clusters and several small subgroups. Because torque values occupied six ordered levels and were concentrated at the predefined target of 35 N·cm, a proportional-odds ordinal logistic regression with patient-clustered robust standard errors was fitted as a sensitivity analysis. Analyses were performed in Python 3.13 using SciPy 1.17.0 and statsmodels 0.14.6.

### 2.7. Use of Generative Artificial Intelligence

ChatGPT (GPT-5.6 Pro; OpenAI, San Francisco, CA, USA) was used to assist with English-language editing, organization of the revision, and minor graphical corrections, including layout, alignment, and label formatting. The tool was not used to generate or modify scientific data, analyses, or study results. All AI-assisted output, as well as the references, interpretations, and final wording, was reviewed and approved by the authors.

## 3. Results

### 3.1. Patient and Implant Characteristics

The 101 implant placements were performed in 55 patients: 30 women and 25 men. Twenty-seven patients received one implant, 20 received two, four received three, one received four, two received five, and one received eight implants. Thus, 28 patients (50.9%) contributed more than one observation. The median number of implants per patient was 2 (Q1–Q3, 1–2), with a range of 1–8. At the first included placement, the mean patient age was 48.5 ± 11.1 years; across implant-level observations, the mean age at placement was 50.5 ± 11.4 years.

At the implant level, 51 placements were performed in women and 50 in men. Sixty-four implants were placed in the maxilla and 37 in the mandible. The maxillary anterior/premolar sector accounted for 45 placements, the maxillary molar sector for 19, the mandibular anterior/premolar sector for 10, and the mandibular molar sector for 27. Eighteen implants had a CHC connection and 83 had a CS connection. Implant lengths were 8 mm (*n* = 14), 10 mm (*n* = 41), 11.5 mm (*n* = 34), and 13 mm (*n* = 12); diameters were 3.2 mm (*n* = 3), 3.5 mm (*n* = 15), 3.75 mm (*n* = 57), 4.2 mm (*n* = 25), and 5.0 mm (*n* = 1). Hard-tissue augmentation accompanied 33 placements: 11 involved a closed sinus-floor elevation, two involved an open sinus-floor elevation, and 20 involved another hard-tissue augmentation procedure. Seven implants were placed immediately and 94 after healing. Table 1 summarizes the cohort.

### 3.2. Distribution of Recorded Insertion Torque

**Figure 1:**
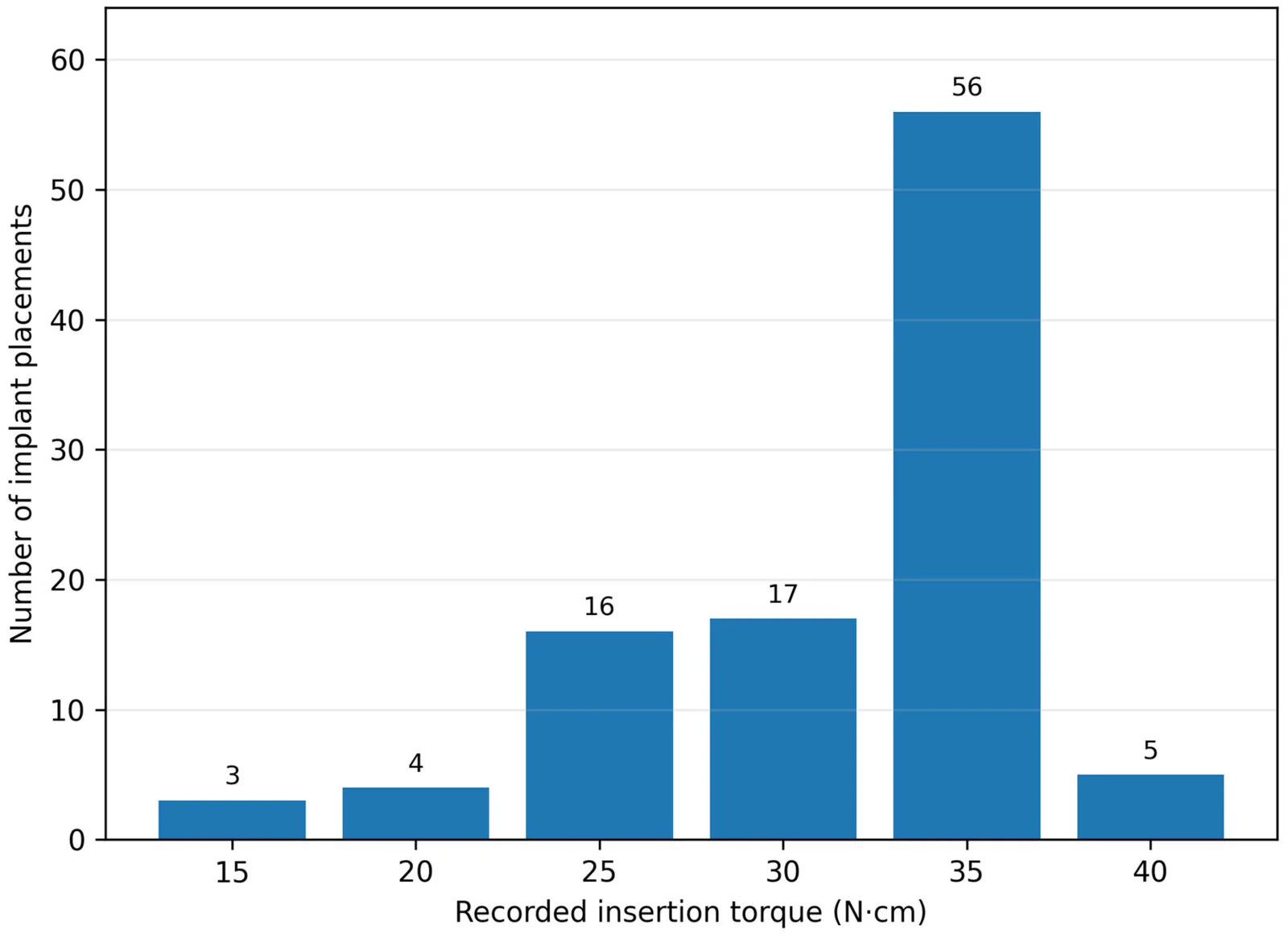
Frequency distribution of the 101 raw recorded insertion-torque values. No values were reconstructed from summary statistics.

Recorded insertion torque was 15 N·cm in three placements, 20 N·cm in four, 25 N·cm in 16, 30 N·cm in 17, 35 N·cm in 56, and 40 N·cm in five. The marked concentration at 35 N·cm, shown in Figure 1, is consistent with the predefined clinical target. Because the Implantmed system and WS-75 L contra-angle permit substantially higher torque, this concentration does not represent the technical upper measurement limit. It does, however, constitute protocol-driven heaping: a charted value of 35 N·cm may indicate attainment of the intended target rather than a continuously resolved peak resistance above that point.

### 3.3. Unadjusted Associations

**Figure 2:**
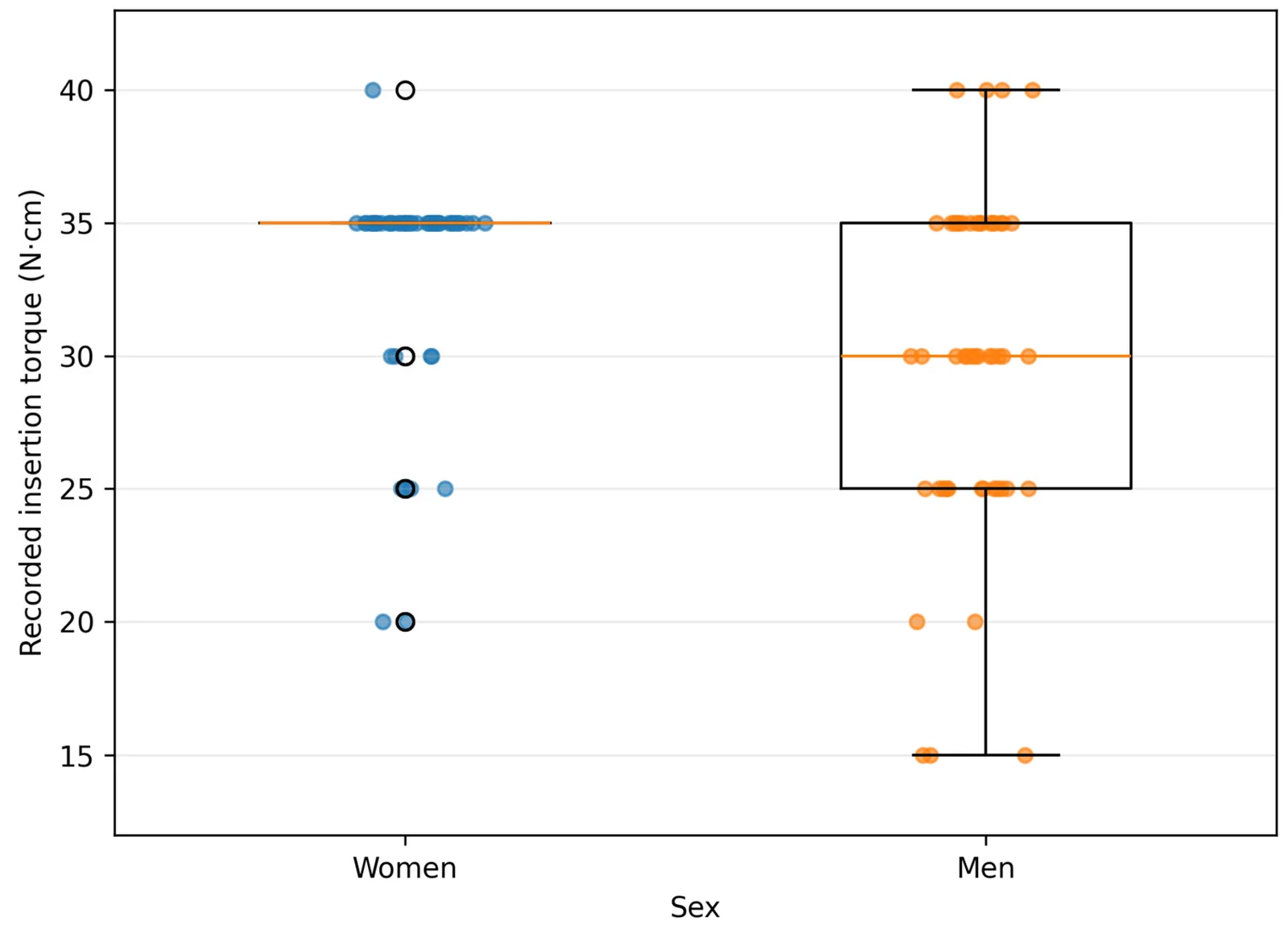
Raw recorded insertion-torque values by sex, overlaid on boxplots calculated directly from the individual observations. Boxes show the interquartile range and centre lines show medians.

In the unadjusted implant-level comparison, placements in women had higher torque than placements in men: median 35 N·cm (Q1–Q3, 35–35) versus 30 N·cm (Q1–Q3, 25–35) (*p* = 0.001). The raw observations and boxplots are presented in Figure 2. Age, jaw, implant length, implant diameter, hard-tissue augmentation, and placement timing were not associated with torque in unadjusted analyses (Table 2).

**Figure 3:**
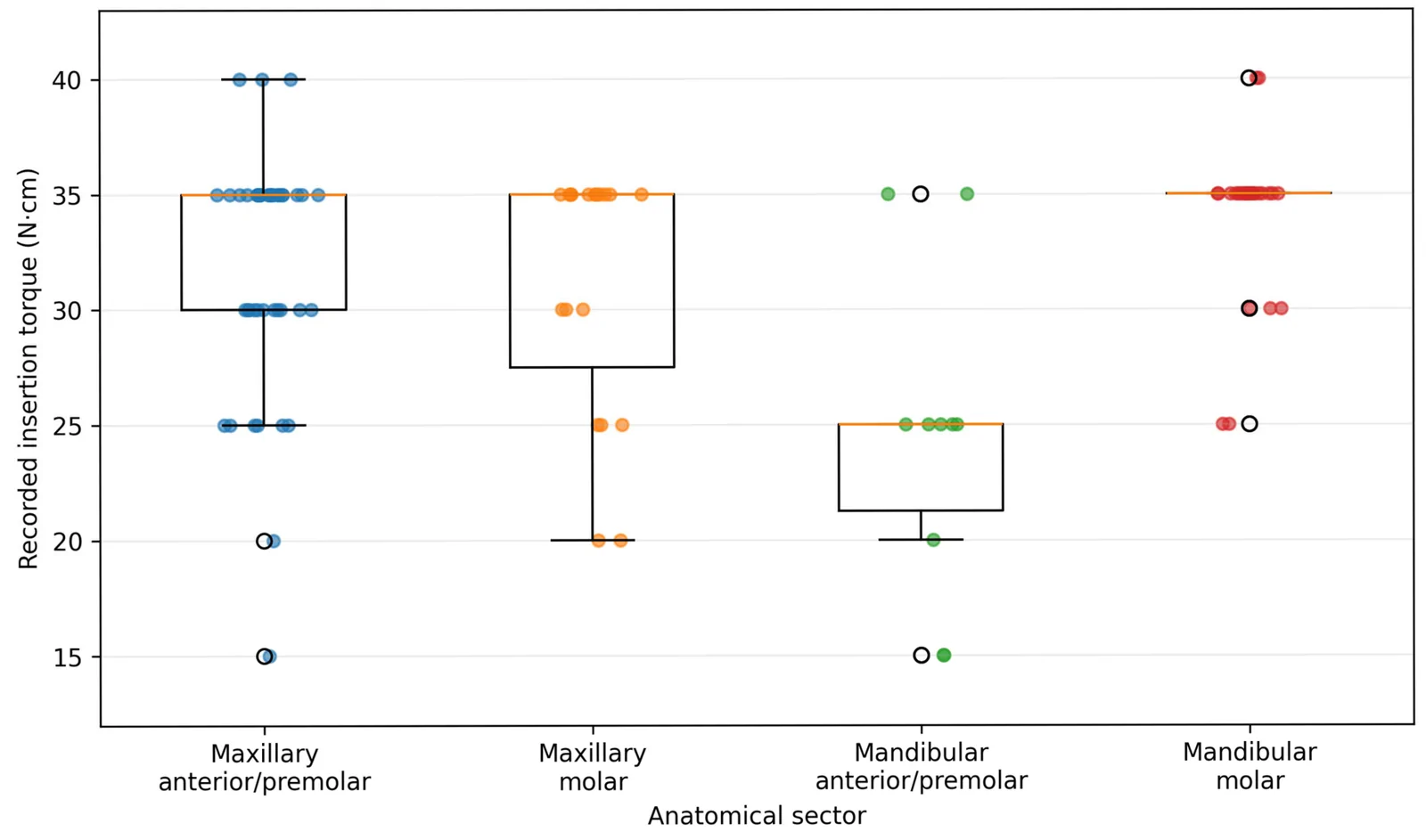
Raw recorded insertion-torque values across the four prespecified anatomical sectors, overlaid on boxplots calculated directly from the individual observations.

The eight detailed tooth regions differed globally (Kruskal–Wallis H = 23.571; *p* = 0.001). Dunn–Holm comparisons showed lower torque in region 31–35 than in 46–48 (adjusted *p* = 0.001) and in 41–45 than in 46–48 (adjusted *p* = 0.020); no other pair remained significant after adjustment. The four-sector comparison was also significant (H = 17.241; *p* < 0.001), with the lowest descriptive values in the mandibular anterior/premolar sector and the highest in the mandibular molar sector (Figure 3).

### 3.4. Patient-Clustered Multivariable Analysis

The generalized estimating equation included all 101 placements and 55 patient clusters; cluster size ranged from one to eight implants, and the estimated exchangeable within-patient correlation was 0.217. After simultaneous adjustment, placements in women remained associated with higher recorded torque (β = 3.25 N·cm; 95% CI 1.20–5.31; *p* = 0.002). Relative to maxillary anterior/premolar sites, torque was lower in mandibular anterior/premolar sites (β = −5.41 N·cm; 95% CI −9.70 to −1.13; *p* = 0.013) and higher in mandibular molar sites (β = 4.02 N·cm; 95% CI 1.60–6.45; *p* = 0.001). Implant length showed a modest positive association of 0.93 N·cm per additional millimetre (95% CI 0.13–1.73; *p* = 0.022). Age, maxillary molar location, implant diameter, hard-tissue augmentation, and immediate placement were not independently associated with torque (Table 3).

The proportional-odds sensitivity model treated 15, 20, 25, 30, 35, and 40 N·cm as ordered categories and used patient-clustered robust standard errors. Placements in women had higher odds of occupying a higher recorded torque category (adjusted odds ratio 3.94; 95% CI 1.29–12.07; *p* = 0.016). The directions of the mandibular sector and implant-length estimates were also consistent with the primary model. This agreement reduces, but does not eliminate, concern arising from the discrete, target-influenced, and clustered distribution of torque values.

## 4. Discussion

The patient-clustered analysis has two important implications. First, the 101 placements arose from 55 individuals and therefore could not be treated as fully independent observations. Second, the adjusted model showed that sex and anatomical sector retained associations with recorded torque, while implant length displayed a modest association that was not apparent in the unadjusted analysis. Implant diameter, age, hard-tissue augmentation, and placement timing were not supported as independent predictors.

The main scientific value lies in examining the mechanical behaviour of a defined titanium implant system within its clinical environment rather than in the sample size alone. All implants shared the Ti-6Al-4V ELI substrate, NanoTec surface, and MultiNeO design principles, while connection type and dimensions varied within that family. This reduces heterogeneity from mixing unrelated implant systems and allows the observed torque to be interpreted as the result of a material–macrogeometry–osteotomy–bone interaction [5,6,7,8,9,10,11,12,28,29,30]. It remains an exploratory clinical-materials study, not a comparison of different alloys or surfaces.

The adjusted association with sex should not be interpreted as proof of an intrinsic biological advantage in women. Skeletal remodelling differs by sex and age, yet those differences are site-specific and do not translate directly into jawbone resistance during implant placement [16,17]. Implant studies have likewise reported inconsistent directions of association: some resonance-frequency studies found higher stability in women, others reported higher values in men, and a systematic review did not establish a simple universal sex effect [18,19,20]. In this cohort, the coefficient may still reflect unmeasured local cortical thickness, trabecular structure, menopausal status, medication use, site selection, or other residual confounding. No sex-specific change in drilling or loading strategy can be recommended from these data.

Anatomical sector produced the largest adjusted differences. Lower values in the mandibular anterior/premolar sector and higher values in the mandibular molar sector are mechanically plausible because cortical thickness, ridge morphology, and the distribution of trabecular bone vary within a jaw [8,13,14]. Nevertheless, the mandibular anterior/premolar sector contained only 10 placements, and the detailed FDI 31–35 region group contained only four placements. The regional estimates are therefore hypothesis-generating and require confirmation in a larger cohort with quantitative cortical measurements.

Implant length was not associated with torque in the unadjusted analysis but became significant after adjustment. This pattern may represent confounding or statistical suppression by sector, diameter, and treatment variables. The estimated effect—approximately 0.9 N·cm per millimetre—was small relative to the overall spread of the data and should not be converted into a deterministic clinical rule. Longer implants can engage more bone, but primary stability also depends on the portion of the implant contacting cortical bone and on how the osteotomy was prepared [5,6,7,8,9,10,11,12,13,14,15]. Diameter was not significant, and the narrow distribution of diameters limited the ability to detect an effect.

Neither hard-tissue augmentation nor immediate placement was independently associated with torque. These null findings do not imply equivalence of procedures. Augmentation comprised several approaches, and only seven implants were placed immediately. Immediate stability depends on residual socket walls and extra-alveolar anchorage, while delayed placement depends on the healed ridge and the selected drilling sequence [21,22,23,24,25,26]. Treatment was chosen clinically rather than randomized, so confounding by indication is unavoidable.

The concentration of 56 observations at 35 N·cm is explained by the clinical protocol: 35 N·cm was the programmed target, not the hardware ceiling. The manufacturer’s official educational material describes this value as an ideal insertion torque and as the minimum indicated for immediate placement or loading, while the W&H system can provide controlled insertion torque up to 80 N·cm [31,32]. The target was therefore selected to preserve flexibility among submerged healing, transgingival healing, and—when the remaining clinical criteria were met—immediate provisionalization. It should not be interpreted as a universal loading rule or as evidence that torque alone determines treatment selection [34]. Because the source records used 5-N·cm categories, values at the target remain semi-quantitative. The cluster-robust ordinal sensitivity analysis was included for this reason, and future prospective studies should export the full motor trace or exact peak value.

Insertion torque remains an intraoperative mechanical observation. The study did not measure resonance frequency, serial implant stability quotient, marginal bone change, implant survival, or histological bone-to-implant contact. It therefore cannot determine whether sex, anatomical sector, or a specific torque value altered osseointegration. The practical implication is narrower: a target of 35 N·cm can preserve several healing and provisionalization options within the described manufacturer-based protocol, but the final choice must also incorporate site anatomy, bone resistance, implant design, prosthetic planning, occlusal control, and patient-level risk factors [3,4,9,23,32,35,36].

Several limitations remain. The retrospective design precludes causal inference, and the absence of a pre-analysis screening log prevents complete assessment of selection bias. Bone quality and cortical thickness guided surgery but were not stored in a standardized case-level form. Smoking, diabetes, osteoporosis, medication exposure, periodontal history, and other systemic variables were unavailable. Four highly experienced surgeons performed the procedures, but operator identifiers were not retained for individual placements, so an operator effect could not be estimated. The sample included small regional and immediate-placement subgroups, and no external validation was available. Finally, the 5-N·cm recording resolution and the predefined target at 35 N·cm limit interpretation of torque as a fully continuous biomechanical measurement.

A prospective study should record patient-level comorbidity data, quantitative cortical thickness, a standardized bone-quality score, operator identity, exact osteotomy sequence, exported motor torque traces, insertion depth, ISQ at placement and follow-up, loading timing, and longitudinal outcomes. Such a design would allow the mechanical contribution of implant geometry and site preparation to be separated more convincingly from patient biology.

## 5. Conclusions

In this exploratory cohort of 55 patients and 101 MultiNeO implant placements, patient-clustered multivariable analysis identified associations between recorded insertion torque and sex, anatomical sector, and implant length. Placements in women had higher adjusted torque, mandibular molar sites had higher values than maxillary anterior/premolar sites, and mandibular anterior/premolar sites had lower values. Age, implant diameter, hard-tissue augmentation, and immediate placement were not independently associated with torque. Because 35 N·cm was a predefined clinical target, the outcome should be interpreted as a recorded value influenced by the protocol rather than as an unrestricted continuous measurement. These results apply to the analysed dataset and do not establish a biological sex effect or a direct relationship with osseointegration. Standardized prospective data on bone quality, cortical thickness, exact torque traces, operator identity, and longitudinal implant outcomes are required before clinical recommendations can be made.

## Figures and Tables

**Table 1:** Patient-, procedure-, and implant-level characteristics.

Level	Variable	Category/Statistic	Value
Patient	Unique patients		55
Patient	Sex	Women	30 (54.55%)
Patient		Men	25 (45.45%)
Patient	Age at first included placement	Mean ± SD, years	48.5 ± 11.1
Patient		Median (Q1–Q3), years	48 (43–55.5)
Patient		Range, years	18–71
Patient	Implants per patient	Mean ± SD	1.84 ± 1.27
Patient		Median (Q1–Q3)	2 (1–2)
Patient		Range	1–8
Patient	More than one implant		28 (50.91%)
Implant	Implant-level observations		101
Implant	Sex of patient	Women	51 (50.50%)
Implant		Men	50 (49.50%)
Implant	Age at placement	Mean ± SD, years	50.5 ± 11.4
Implant		Median (Q1–Q3), years	51 (45–58)
Implant		Range, years	18–71
Implant	Jaw	Maxilla	64 (63.37%)
Implant		Mandible	37 (36.63%)
Implant	Anatomical sector	Maxillary anterior/premolar	45 (44.55%)
Implant		Maxillary molar	19 (18.81%)
Implant		Mandibular anterior/premolar	10 (9.90%)
Implant		Mandibular molar	27 (26.73%)
Implant	Connection	CHC	18 (17.82%)
Implant		CS	83 (82.18%)
Implant	Length	Mean ± SD, mm	10.58 ± 1.44
Implant		Median (Q1–Q3), mm	10 (10–11.5)
Implant		Range, mm	8–13
Implant	Diameter	Mean ± SD, mm	3.82 ± 0.28
Implant		Median (Q1–Q3), mm	3.75 (3.75–4.20)
Implant		Range, mm	3.20–5.00
Implant	Hard-tissue augmentation	No	68 (67.33%)
Implant		Yes	33 (32.67%)
Implant	Placement timing	Delayed	94 (93.07%)
Implant		Immediate	7 (6.93%)
Implant	Recorded insertion torque	Mean ± SD, N·cm	31.63 ± 5.57
Implant		Median (Q1–Q3), N·cm	35 (30–35)
Implant		Range, N·cm	15–40

CHC, Conical Narrow Connection; CS, Conical Standard Connection; SD, standard deviation; Q1, first quartile; Q3, third quartile. Percentages at patient level use 55 as the denominator; percentages at implant level use 101.

**Table 2:** Unadjusted associations with recorded insertion torque.

Variable	Comparison/Analysis	Statistic	*p*-Value	Result
Sex	Women vs. men	U = 836.0	0.001	Higher torque in placements in women
Age groups	≤45, 46–55, >55 years	H = 0.205	0.902	Not significant
Age	Continuous	ρ = −0.001	0.990	Not significant
Jaw	Maxilla vs. mandible	U = 1152.0	0.806	Not significant
Implant length	Continuous	ρ = 0.079	0.431	Not significant
Implant diameter	Continuous	ρ = −0.107	0.289	Not significant
Hard-tissue augmentation	No vs. yes	U = 1128.5	0.962	Not significant
Placement timing	Delayed vs. immediate	U = 316.0	0.854	Not significant
Detailed tooth region	Eight FDI region groups	H = 23.571	0.001	Global difference
Anatomical sector	Four aggregated sectors	H = 17.241	<0.001	Global difference

FDI, Fédération Dentaire Internationale; U, Mann–Whitney U statistic; H, Kruskal–Wallis statistic; ρ, Spearman rank correlation. Pairwise regional comparisons used Dunn’s test with Holm adjustment.

**Table 3:** Patient-clustered Gaussian generalized estimating equation for recorded insertion torque.

Predictor	Adjusted β (N·cm)	Robust SE	95% CI	*p*-Value
Female sex (vs. male)	3.25	1.05	1.20 to 5.31	0.002
Age, per 10 years	0.50	0.56	−0.59 to 1.60	0.367
Maxillary molar sector	−0.52	1.56	−3.58 to 2.54	0.739
Mandibular anterior/premolar sector	−5.41	2.19	−9.70 to −1.13	0.013
Mandibular molar sector	4.02	1.24	1.60 to 6.45	0.001
Implant length, per mm	0.93	0.41	0.13 to 1.73	0.022
Implant diameter, per mm	−2.35	2.21	−6.67 to 1.98	0.288
Hard-tissue augmentation	0.41	1.24	−2.02 to 2.84	0.743
Immediate placement	0.99	1.37	−1.70 to 3.69	0.470

CI, confidence interval; SE, standard error. Reference categories: male sex, maxillary anterior/premolar sector, no hard-tissue augmentation, and delayed placement. β values represent adjusted mean differences in recorded torque. Robust standard errors account for clustering within 55 patients.

## Data Availability

The original contributions presented in this study are included in the article. Further inquiries can be directed to the corresponding author.

## Abbreviations

The following abbreviations are used in this manuscript:

Abbreviation	Meaning
CHC	Conical Narrow Connection
CI	Confidence interval
CS	Conical Standard Connection
GEE	Generalized estimating equation
ISQ	Implant stability quotient
IT	Insertion torque
RFA	Resonance frequency analysis
SD	Standard deviation
